# Tandem fecal microbiota transplantation cycles in an allogeneic hematopoietic stem cell transplant recipient targeting carbapenem-resistant Enterobacteriaceae colonization: a case report and literature review

**DOI:** 10.1186/s40001-021-00508-8

**Published:** 2021-04-28

**Authors:** Fengqin Su, Yi Luo, Jian Yu, Jimin Shi, Yanmin Zhao, Mengni Yan, He Huang, Yamin Tan

**Affiliations:** 1 Bone Marrow Transplantation Center, The First Affiliated Hospital, Zhejiang University School of Medicine, 79 Qingchun Road, Hangzhou, 310003 China; 2 Institute of Hematology, Zhejiang University, Hangzhou, Zhejiang People’s Republic of China; 3 Hematology Department, The Cancer Hospital of the University of Chinese Academy of Sciences (Zhejiang Cancer Hospital), Institute of Basic Medicine and Cancer (IBMC), Chinese Academy of Sciences, 1 Banshan East Road, Hangzhou, 310022 Zhejiang China

**Keywords:** Carbapenem-resistant Enterobacteriaceae colonization, Fecal microbiota transplantation, Gut microbiota, Hematopoietic stem cell transplantation, Multidrug-resistant bacteria

## Abstract

**Background:**

Due to limited antibiotic options, carbapenem-resistant Enterobacteriaceae (CRE) infections are associated with high non-relapse mortality after allogeneic hematopoietic stem cell transplantation (allo-HSCT). Also, intestinal CRE colonization is a risk factor for subsequent CRE infection. Several clinical studies have reported successful fecal microbiota transplantation (FMT) for the gut decontamination of a variety of multidrug-resistant bacteria (MDRB), even in immunosuppressed patients. Similarly, other studies have also indicated that multiple FMTs may increase or lead to successful therapeutic outcomes.

**Case presentation:**

We report CRE colonization in an allo-HSCT patient with recurrent CRE infections, and its successful eradication using tandem FMT cycles at 488 days after allo-HSCT. We also performed a comprehensive microbiota analysis. No acute or delayed adverse events (AEs) were observed. The patient remained clinically stable with CRE-negative stool culture at 26-month follow-up. Our analyses also showed some gut microbiota reconstruction. We also reviewed the current literature on decolonization strategies for CRE.

**Conclusions:**

CRE colonization led to a high no-relapse mortality after allo-HSCT; however, well-established decolonization strategies are currently lacking. The successful decolonization of this patient suggests that multiple FMT cycles may be potential options for CRE decolonization.

**Supplementary Information:**

The online version contains supplementary material available at 10.1186/s40001-021-00508-8.

## Background

Carbapenem-resistant Enterobacteriaceae (CRE) pose a significant threat to global health due to limited antibiotic options, especially for immunocompromised patients, such as solid organ and hematological transplant recipients. In a nationwide Italian retrospective survey, CRE infection cases were reported in 53.4% of hematopoietic stem cell transplant (HSCT) centers, involving 2% of allogeneic-HSCT (allo-HSCT) recipients, with 39.2% of allo-HSCT patients colonized by subsequent CRE infections [1]. For hematological patients, CRE colonization is a known risk factor for subsequent CRE infections [2, 3]. Previous research has shown that CRE infection-related fatality rates, ranging from 52.2 to 64.4%, occur in hematologic malignancies [4, 5].

Fecal microbiota transplantation (FMT) is a novel therapeutic strategy and is recommended by several international guidelines as an effective treatment option for recurrent Clostridium difficile infection (CDI) [6–9]. FMT is also being explored as a potential therapy for other conditions, including inflammatory bowel disease [10, 11], irritable bowel syndrome[12, 13], graft-versus-host disease (GVHD) [14–17], the decolonization of multidrug-resistant bacteria (MDRB) [18–20], and several ongoing clinical trials; NCT04711967 (prospective study of FMT for acute intestinal GVHD after allo-HSCT) and NCT03678493 (a study of FMT in patients with AML allo-HSCT in recipients). FMT efficacy for MDRB decolonization is believed to be secondary to the transfer of organisms that restore microbiome diversity and provide colonization resistance [21]. Previous studies have suggested that multiple FMTs may increase the chance of successful therapeutic outcomes [22–24]. Thus, we hypothesized that multiple FMTs may exert significant effects on CRE decolonization in a manner similar to CDI. Therefore, we used tandem FMT cycles to successfully eradicate gut CRE in an allo-HSCT recipient with recurrent CRE infections. We also analyzed the gut microbiota to provide structural insights on ongoing microbiome reconfiguration.

## Case presentation

**Fig. 1:**
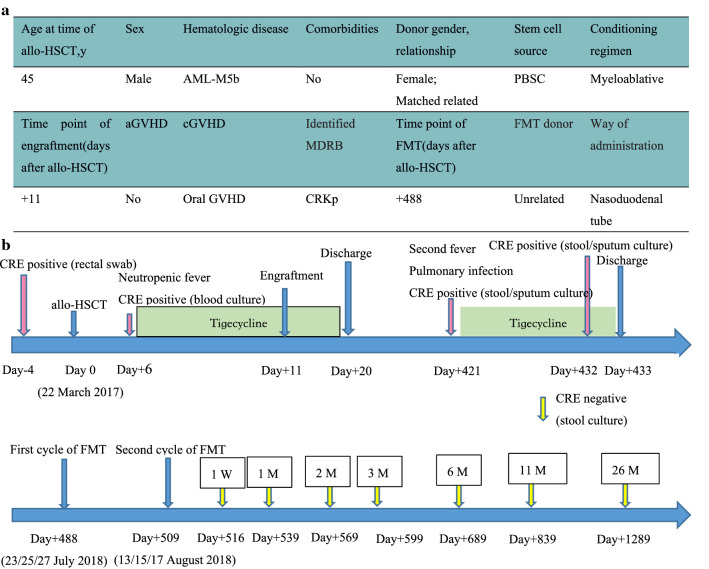
Characteristics of the patient. **a** Characteristics of the patient undergoing FMT before and after allo-HSCT. **b** The time line scale in days from the beginning of allo-HSCT allo-HSCT, allogeneic hematopoietic stem cell transplantation; *AML* acute myeloid leukemia, *PBSC* peripheral blood stem cells, *GVHD* graft-versus-host disease, *CRKp* carbapenem-resistant *Klebsiella pneumonia,* *MDRB* multidrug-resistant bacteria, *FMT* Fecal microbiota transplantation, *CRE* carbapenem-resistant Enterobacteriaceae, *W* week, *M *month

A 45-year-old man with acute myeloid leukemia (AML-M5b) was subject to human leukocyte antigen-haploidentical relative HSCT for persistent minimal residual disease (MRD) (Fig. 1a). Apart from AML, the patient did not have any potentially relevant pre-existing conditions or medical treatments that may have impacted on bacterial colonization, clearance, or drug tolerance. The conditioning regimen comprised busulfan (0.8 mg/kg/q6h), cyclophosphamide (60 mg/kg/d), and semustine (250 mg/m^2^/d). Cyclosporin A, methotrexate, and mycophenolate mofetil were used for GVHD prophylaxis, and levofloxacin and cefotaxime/sulbactam were given for antimicrobial prophylaxis. Prior to conditioning therapy, carbapenem-resistant *Klebsiella pneumoniae* (CRKp) colonization, which has a high level of resistance to most routine antibiotics except for tigecycline (Additional file 1: Figure S1), was identified during routine rectal screening, while no decolonization strategies were performed. Six days after allo-HSCT, the patient developed neutropenic fever and the organism that grew in the blood culture was identified as CRKp which with the same resistance pattern as the previous one. CRKp bacteremia was successfully controlled by tigecycline. Hematopoietic stem cells were engrafted on day +11 and the patient discharged on day +20. During regular follow-up, the patient was in remission, but suffered chronic oral GVHD as it had not responded well to prednisone and tacrolimus. Thus, the patient remained in a very poor nutritional state, weighing approximately 45 kg (height = 168 cm).

A second fever episode was accompanied by chills, cough, and expectoration on day +421. On this occasion, lung computerized tomography (CT) suggested pulmonary infection, and sputum and stool cultures were both positive for CRKp. This confirmed previous susceptibility tests, although blood cultures were negative. Infection was controlled by administering an antibiotic therapeutic regimen, including tigecycline. However, sputum and stool cultures remained positive for CRKp, indicating persistent CRKp colonization. Based on the same resistance patterns, we speculated that they were the same strain. Moreover, a previous study indicated that most bacteremia cases originated from the gut [25, 26]. Thus, FMT was planned to reduce the risk of infection and improve future quality of life.

## Methods

Frozen microbiota stocks were generated by a non-profit stool bank (MedBiome, Xian, China). The unrelated donor was a 23-year-old male volunteer, who was carefully screened using our inclusion and exclusion criteria (Additional file 2: Table S1) [27]. Prior to donation, the donor provided informed written consent and was provided with a nutritious, balanced diet, without seafood, spices, and unclean food for 3 days. This donor contributed independently two fecal samples for this study.

Donor fecal microbiota was prepared by the stoolbank in a Good Manufacturing Practice (GMP)-level laboratory and workflow. Fresh feces were purified using a newly developed automatic purification system (GenFMTer, MedBiome, Xian, China), which ensured increased quality control when compared with manual sample preparation. The process also significantly reduced FMT-related adverse events (AEs) by removing undigested food residues, fungi, parasite eggs, and some small particles [28]. The precipitate was removed from the washed fecal suspension by centrifugation, followed by washing three times in sterile saline, and re-centrifugation. Thus, crudely purified fecal microbiota precipitate was obtained, and approximately 30–40 ml precipitate was resuspended in 30% sterile glycerin, packaged in each of three plastic bottles, and stored at − 80 °C. Each time, the laboratory supplied the three frozen microbiota stocks for one cycle which were transported to the clinic on dry ice before the cycle.

Prior to FMT, the suspension from one plastic bottle was thawed at 37 °C and purified by repeated centrifugation. The precipitate was diluted to 200 ml (except on one occasion when it was diluted to 250 ml) in sterile saline. It was then stirred, and was ready for use when completely homogenized.

Before the procedure, the patient provided written informed consent. To reduce the risk of failure, we performed tandem FMT cycles.

Fecal microbiota preparations were delivered to the intestine over 2–4 min using a nasoduodenal tube (localized using X rays). The patient then fasted for 2 h after which he was permitted to eat and drink normally. This procedure was repeated three times every other day for each course. Two courses were performed with a 17 day interval (23/25/27 July 2018 and 13/15/17 Aug 2018) (Fig. 1b). FMT was initiated at day +488, while the patient was under prednisone immunosuppressive therapy, and no antibiotics were taken since his last discharge from hospital. The patient was observed for a few hours at the hospital after each procedure. He was regularly followed up in the outpatient clinic using stool culture samples.

For stool analysis, we performed the following; prior to FMT, 1 week and 3 weeks after the first cycle, and 1 week, 3 months, and 11 months after the second, we collected stool samples, extracted DNA, and analyzed 16 s rDNA amplicons using an Illumina Miseq (Genetalks, Changsha, China). Raw sequence data were analyzed using Quantitative Insights Into Microbial Ecology software (QIIME, version 1.9.1), and visualized by R (version 3.5.1, R Foundation for Statistical Computing, Vienna, Austria). Microbial diversity was estimated using the Shannon diversity index.

## Results

The patient tolerated tandem FMT cycles without AEs during the initial process and follow-up period (26 months). Throughout follow-up after FMT termination, and for the next 26 months (between August 2018 and October 2020), stool cultures were CRE negative at 1 week, 1 month, 2 months, 3 months, 6 months, 11 months, and 26 months, and the patient remained clinically stable. The only fever episode occurred at 12 months, and included a positive sputum culture for the same CRKp as previously observed, but lung CT was clear and blood and stool cultures were negative. Empirical treatment with piperacillin/tazobactam did not control the fever, but it was quickly resolved by successive meropenem administration, suggesting that the CRKp may not have been the causative pathogen this time. A significant improvement was observed in oral chronic GVHD, and the patient gained approximately 5 kg over 8 months following FMT.

**Fig. 2:**
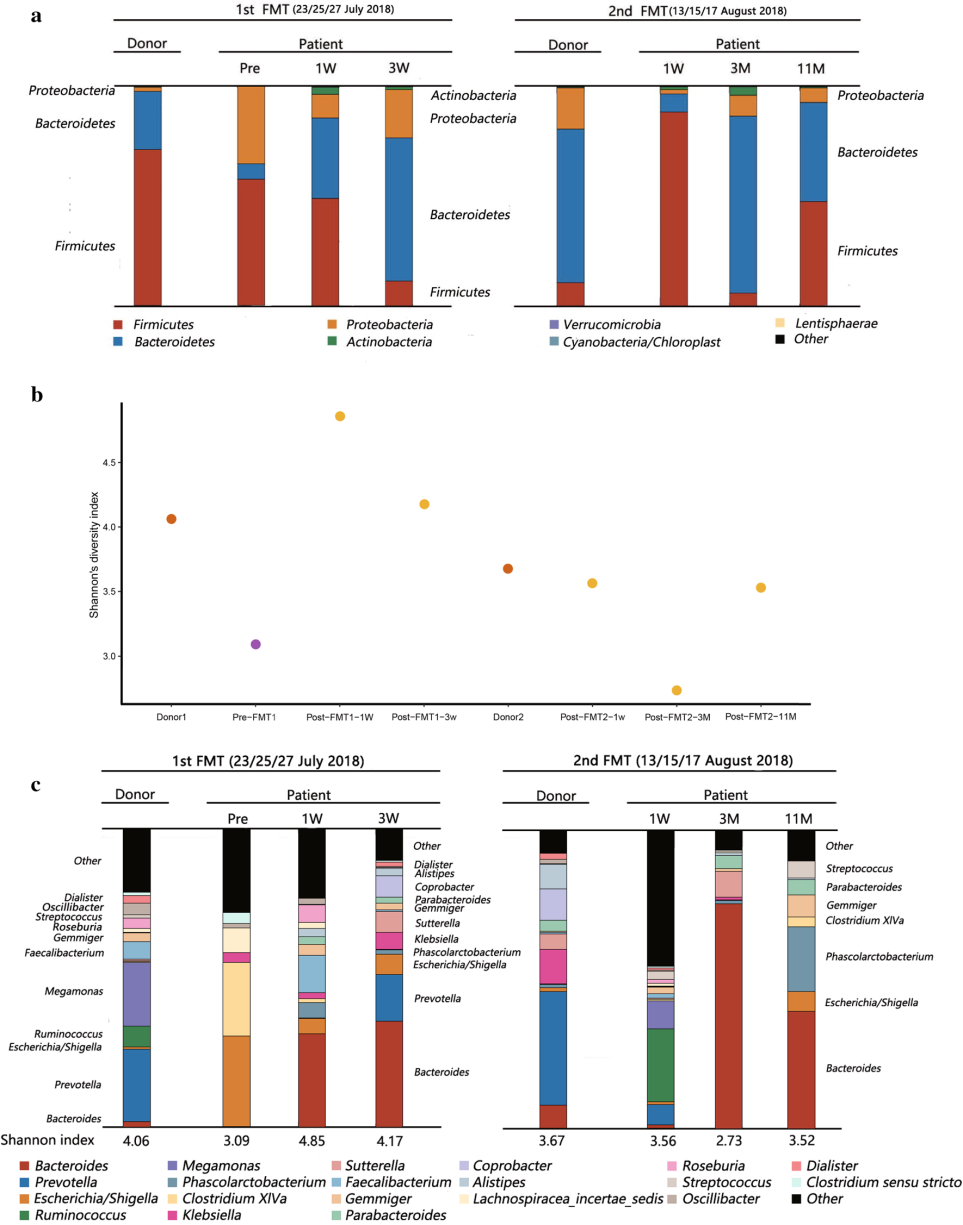
Microbiota analysis. **a** Changes in bacterial community composition on phylum level. **b** Change in Shannon’ diversity index. **c** Changes in bacterial community composition on genus level. The fecal sample obtained 2 days before the initiation of the first FMT was analyzed as ‘pre’ data. The time points ‘1 W’, ‘3 W’, ‘3 M’, and ‘11 M’ indicate periods from the end of the first or second cycle of FMT; *W* week, *M* month

To identify correlations between fecal microbiota and clinical benefits, we evaluated changes in bacterial microbiota before and after FMT cycles using 16 s rDNA sequencing (Fig. 2). Prior to FMT, we detected pathobionts of the Proteobacteria phylum, more notably *Escherichia/Shigella* and *Klebsiella*, which accounted for > 33% of the whole community, and was far higher than healthy individuals [29]. Eleven months after tandem FMT cycles, with the expansion of the protective phylum, Bacteroidetes, the patients’ microbial composition was eventually dominated by Firmicutes and Bacteroidetes, similar to normal commensal patterns (Fig. 2a) [30]. Bacteroidetes were associated with protection against Enterococcus domination [31], improved gut GVHD [32, 33], protection against CDI [34], and protection against Gram-negative blood infections [35], which were mainly associated with short-chain fatty acids’ (SCFAs) production. In contrast, the outgrowth of opportunistic pathogens belonging to Proteobacteria had been linked to increased treatment-related mortality, including GVHD, infections, and organ failure after allo-HSCT [36]. In a recent study, recolonization with microbiota containing anaerobic *Prevotella* species as a keystone genus was correlated with CRE decolonization [37]. In our case, the donor’s two fecal samples both contained significant *Prevotella* levels which are not ordinarily detected in patient’s fecal microbiota before FMT. Also, levels were significant 3 weeks after the first FMT cycle and remained for 1 week after the second cycle (Fig. 2c). Although levels disappeared 3 weeks after the second FMT cycle, our observations suggest that *Prevotella* may play an important role in CRE decolonization. Fecal microbiota diversity (Shannon diversity index) also increased from 3.09 to 3.52 (final measurement) (Fig. 2b).

**Fig. 3:**
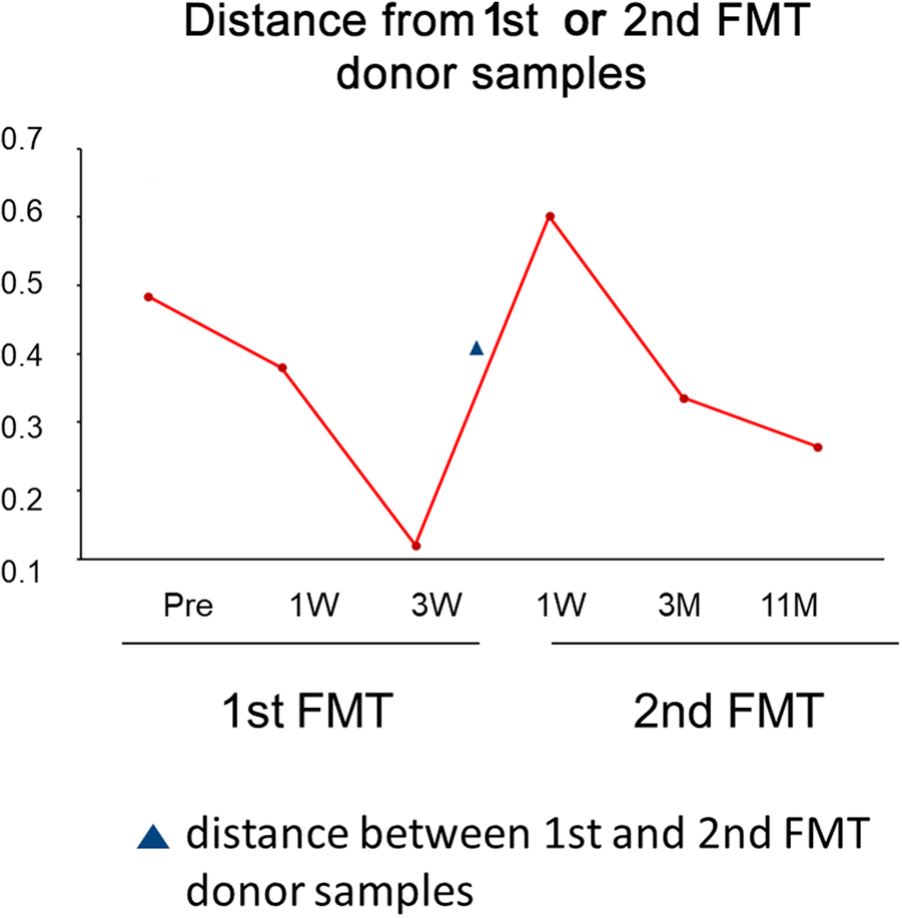
Weighted UniFrac distance analysis: Distance from the donor fecal microbiota of the first or second FMT. The fecal sample that was obtained 2 days before the initiation of the first FMT was analyzed as “pre” data. The time points “1 W,” “3 W,” “3 M,” and “11 M” indicate periods from the end of the first or second cycle of FMT; *W* week, *M* month

We also analyzed differences between the patient’s fecal microbiota and the first or second donor fecal sample using UniFrac distance analysis (Fig. 3). The difference was dramatically increased after the second FMT cycle. This may have been due to the difference between the donor’s two samples. The patient’s fecal microbiota closely matched the donor’s fecal microbiota after both cycles, suggesting that the grafted samples contributed to diversity recovery.

## Discussion and conclusions

Previous reports have shown that colonization with MDRB, including CRE and vancomycin-resistant enterococci (VRE), exerts a negative impact on overall survival after allo-HSCT due to a higher incidence of infection, especially in patients with lower gut microbiota diversity [38]. Healthy gut microbiota can prevent invading pathogens from colonizing the intestinal tract, a phenomenon known as colonization resistance. This process is underpinned by several mechanisms, including competition for metabolic and physical niches, production of inhibitory metabolites, and interaction with the host immune system [39]. However, major factors including conditional chemotherapy and/or irradiation, antibiotic therapy, GVHD, mucositis, changes in diet, and infection (e.g., *C. difficile*) can alter microbiota homeostasis in allo-HSCT, and cause loss of this colonization resistance in allo-HSCT recipients [40, 41]. We therefore speculated that gut composition and diversity restoration by FMT could benefit allo-HSCT recipients and clear CRE from the gut.

The literature suggests that spontaneous CRE decolonization takes time. In a retrospective multicenter study conducted in two different tertiary care hospitals, the spontaneous decolonization of CRE and VRE occurred within the first 30 days in 16.4% of cases, and 48.2% after 3 months, with a median follow-up of 96 days (0–974) [42]. In addition, Haverkate et al. also reported that only 17% of long-term acute care hospital patients lost CRKp colonization within 4 weeks, and approximately 50% were still carriers after 9 months [43]. A previous meta-analysis reported that the rate of spontaneous CRE decolonization was only 23.3%, and a significant proportion of carriers (35.2%) were still colonized up to 12 months later [44].

The most common strategy for gut CRE decolonization is oral, non-absorbable antibiotics, including gentamicin, colistin, or polymyxin E, which must achieve sufficiently high concentrations in the digestive tract to inhibit bacterial growth. The decolonization rate of these antibiotics ranges between 37.5 and 71% [45–47], and up to 66% in HSCT recipients [48]. These data were significantly higher than the spontaneous decolonization group. Previous studies have also shown that antibiotic therapies are associated with dramatic increases in antibiotic-resistant genes, and may increase microbial resistance in the future [49, 50]. Furthermore, due to long-term hospitalization, low functional patient status, and gut microbiota dysbiosis, even after successful decolonization, allo-HSCT recipients are more likely to reacquire colonization from other patients or the environment [51].

Several case reports and small-sample clinical studies have reported the beneficial effects of FMT towards MDRB decolonization in immunosuppressed patients with blood disorders. A prospective, single-center study by Bilinski et al. showed the complete eradication of MDRB in 15 of 20 patients with blood disorders after FMT, with a higher abundance of Barnesiella species, Bacteroides, and Butyricimonas in responders [52]. Of note, six patients received FMT after allo-HSCT. Similarly, Battipaglia et al. successfully eradicated CRE/VRE after FMT in 7 of 10 patients with hematologic malignancies, before or after allo-HCST [53]. More recently, Merli et al. showed that MDRB decolonization was achieved in four of five (80%) pediatric patients before allo-HSCT by FMT, within 1 week [54]. These reports supported not only FMT efficacy but also demonstrated that FMT in allo-HSCT settings was safe and tolerable. Furthermore, some studies have also suggested a significant reduction in antibiotic-resistant gene carriage in recipient microbiota following FMT for CDI [55–57].

However, some studies have observed mixed conclusions. Sohn et al. performed FMT to eradicate long-term VRE colonization in three patients; however, only one patient was cleared at 15 weeks after FMT [58]. Several reasons may be responsible for these observations. First, small-sample sizes may have contributed to the different results. Second, age may be a factor affecting FMT results, as outlined in some studies [59, 60]. The patients in the study by Sohn et al. were older (median age was 74.7 vs. 51 years in Bilinski et al. vs. 48 years in Battipaglia et al. vs. 11.4 years in Merli et al.). In a prospective comparative study, Dinh et al. observed that VRE clearance after FMT appeared to be quicker than CRE clearance (87.5% vs. 50%)[61]. In addition to factors discussed by these authors, the age differences between the CRE and VRE groups (median age was 66 years for VRE vs. 73.5 years for CRE) may also have contributed to the different results. This may have been due to longer hospitalization and more underlying significant comorbidities in elderly patients. Other factors including stool donors, administration methods, antibiotic use before or after FMT, and the number and frequency of administrations may have impacted FMT outcomes. As a consequence, multi-center and randomized-controlled trials are ongoing to evaluate the true impact of FMT for MDRB eradication; NCT04181112 (fecal transplant for MDRO decolonization) and NCT04759001 (FMT for the decolonization of carbapenem-resistant Enterobacteriaceae).

In our case, AEs were not observed; however, FMT-related AEs have been previously reported in other studies. Recently, a systematic review summarized the global incidence of FMT-related AEs between 2000 and 2020 [62]. Most were mild, moderate, and self-limiting, and the most frequently reported AEs were diarrhea (10%) and abdominal discomfort/pain/cramping (7%), which may have been due to most patients receiving FMT with impaired intestinal mucosal barriers and severe inflammation. FMT-related serious adverse events (SAEs), including infections and deaths were reported in 1.4% of patients, all of whom had mucosal barrier injuries. For MDRB decolonization in patients with hematologic malignancies, the main AEs were mild and transient gastro-intestinal symptoms, including diarrhea, abdominal discomfort, nausea, bloating, and constipation, with no major AEs reported [63]. We have summarized all recently reported FMT-related AEs in allo-HSCT patients (Table 1). In line with the literature, most AEs were mild, transient, and self-limiting, and the most frequently reported AEs were diarrhea (22.79%) and abdominal pain/discomfort/bloating/urgency (7.35%). The diarrhea incidence was higher in allo-HSCT patients, especially in those with neutropenia. Other AEs including nausea, vomiting, and pharyngolaryngeal pain were reported in 2.21% of FMT procedures, respectively. Overall, the FMT has shown an excellent safety profile. However, in a most recent study by Bilinski et al., higher rate of SAEs, including septic shock, sepsis, and norovirus-mediated GI tract infection were observed, which may have been due to the poor general performance status of study patients [64]. Moreover, it was observed that a worse general performance status tended to correlate with more frequent complications. Importantly, none of these events resulted in death. In addition, several reports have indicated no significant differences in SAE rates between immunocompromised and immunocompetent patients [65, 66]. Thus, immunosuppression may not be a contraindication for FMT; however, the procedure still should be used with caution, especially in patients with a low-performance status.

**Table 1:** Studies of FMT in allo-HSCT patients

Authors	Study type	Number of patients	Age	Sex	Number of allo-HSCT patients	Immunocompromission	Outcome	Route of FMT Administration	Times of FMT	FMT-related adverse events (number of times)
FMT for GVHD treatment										
Kakihana, 2016 Japan [14]	Pilot study	4	46 (42–64)	2 M 2F	1	No mention	3 complete response ,1 partial response	Nasoduodenal tube	7	Abdominal pain (4) Diarrhea (3) Pharyngolaryngeal pain (3) Nausea (1) Belch (1)
Spindelboeck, 2017 Austria [17]	Retrospective case series	3	60 (53–61)	1 M 2F	3	No mention	2 complete resolution, 1 partial resolution	Colonoscopy	9	None
Qi, 2018 China [15]	Pilot study	8	35.6 (20–48)	3 M 5F	8	No mention	All the patients achieved clinical symptomatic remission	Nasoduodenal tube	12	None
Kaito, 2018 Japan [16]	Case report	1	21	F	1	No mention	Bacterial diversity was restored, with improvement of diarrhea	Oral capsule		Transient fever Herpes zoster
Mao, 2020 China [67]	Case report	1	31	M	1	No mention	Intestinal aGVHD was controlled and did not recur again	Oral capsule	2	None
Bilinski, 2021 Poland [63]	Prospective multicenter study	13	41.5 (23–66)	9 M 4F	13	No mention	Overall response rate reached 62.5% (10/16)	Nasoduodenal tube	16	Septic shock (1) Sepsis (1) Norovirus-mediated GI tract infection (1)
FMT for MDRB decolonization										
Bilinski, 2017 Poland [51]	Prospective single-center study	20	51 (22–77)	14 M 6F	6	8 patients with neutropenia (< 1.8 × 10^9^ neutrophils/L), but not severe neutropenia (< 0.5 × 10^9^ /L)	15/20 (75%) decolonization	Nasoduodenal tube	25	Vomiting (1) Diarrhea (25)
Innes, 2017 UK [68]	Case report	1	63	M	1	FMT was administrated 2 weeks before allo-HSCT	All kinds of MRD were decolonized	Nasogastric tube	1	Mild, self-limited nausea, loose stool and abdominal discomfort
Battipaglia, 2019 France [52]	Retrospective single-center study	10	48 (16–64)	4 M 6F	6 (4 patients before allo-HSCT)	Neutrophil count was > 1 × 10^9^ /L in all patients but one who had a neutrophil count of 0.17 × 10^9^ /L	7/10 (70%) decolonization	Enema (10) Nasogastric tube (3)	13	Constipation (1) Diarrhea (2)
FMT for rCDI treatment										
Webb, 2016 USA [69]	Retrospective case series	7	43 (33–51)	4 M 3F	7	No mention	7/7 (100%) without rCDI	Nasojejunal tube (6) Colonoscopy (2)	8	Mild self-limited GI discomfort/bloating (2) Self-limited chills (1) Bloating and urgency (2) (one patient was suspected of having small intestinal bacterial overgrowth) Self-limited right upper quadrant pain (1)
Moss, 2017 USA [70]	Retrospective, case series	8	56 (38–71)	3 M 5F	6 (two patients after auto-HSCT)	No mention	8/8 (100%) patients with resolution of rCDI	Oral capsule	8	Vomiting (1)
Bluestone, 2018 USA [71]	Retrospective, case series	3	8 (2–12)	2 M 1F	3	No mention	1/3 (33.3%) patient without rCDI	Gastric tube (6) Colonoscopy (1) Gastrojejunal tube (1)	8	Nausea and retching (1) Vomiting (1)
FMT for intestinal microbiome reconstruction										
DeFilipp, 2018 USA [72]	Open-label single-group pilot study	13	63 (26–71)	6 M 7F	13	FMT capsules were administered no later than 4 weeks after neutrophil engraftment	FMT led to early expansion of microbiome diversity	Oral capsule	13	Severe abdominal pain (1)
Taur, 2019 USA [73]	Randomized-controlled clinical trial	14	52.5 (32–71)	6 M 8F	14	FMT was administrated a median of 18 (8–27) days after engraftment	Diversity and composition of gut microbiota were restored	Enema	14	None
The total number of FMT times									136	
Diarrhea									*n* = 31 (22.79%)	
Abdominal pain/discomfort/bloating/urgency									*n* = 10 (7.35%)	
Nausea									*n* = 3 (2.21%)	
Vomiting									*n* = 3 (2.21%)	
Pharyngolaryngeal pain									*n* = 3 (2.21%)	
Others (belch/fever/herpes zoster/septic shock/sepsis/norovirus-mediated GI tract infection/constipation/chills/right upper quadrant pain)									*n* = 9 (6.62%)	

*allo-HSCT* allogeneic hematopoietic stem cell transplantation, *FMT* fecal microbiota transplantation, *GVHD* graft-versus-host disease, *M* male, *F* female, *aGVHD* acute graft-versus-host disease, *GI* gastrointestinal, *MDRB* multidrug-resistant bacteria, *rCDI* recurrent *Clostridium difficile* infection, *auto-HSCT *autogeneic hematopoietic stem cell transplantation

DeFilipp et al. described two immunocompromised recipients with bacteremia from drug-resistant *Escherichia coli* in donor stool; one recipient with hematologic malignancy who had received FMT capsules on day 3 and day 4 before allo-HSCT died [74]. This study generated an SAE safety alert due to MDRB transmission via FMT, especially in immunocompromised patients. Therefore, standardized donor-screening protocols, included MDRB detection, are urgently required in the field. To avoid potential risks from FMT, probiotic cocktails could be used as alternative strategies. Nagpal et al. developed a novel human-origin probiotic cocktail containing five *Lactobacillus* and five *Enterococcus* strains from healthy infant gut which modulated the fecal microbiome and enhanced SCFAs in mouse gut and human feces [75]. Similarly, Ahmadi et al., in an aged mouse model, showed the probiotic cocktail counteracted metabolic syndrome, and deeply reshaped the gut microbiota [76]. However, according to our literature review, this novel strategy has not been used to decolonize MDRB, and therefore, further study is warranted.

In our case, tandem FMT cycles resulted in successful CRE decolonization of the gut, and a concomitant improvement in quality of life. As we observed only one patient, limited conclusions may be drawn; however, these data suggest that multiple FMTs may be viable options for CRE decolonization. Additionally, our 26-month follow-up was longer than all previous studies. Many aspects of FMT remain unknown and the long-term consequences are unclear, and therefore, standardized and harmonized studies are required to properly evaluate FMT as a promising clinical strategy for microbiome-related disorders.

## Supplementary Information

**Additional file 1: Figure S1.** Antibiogram of the cultured CRKp. *R* resistant, *S* sensitive.

**Additional file 2: Table S1.** Protocol of donor screening in MedBiome. *IBD* inflammatory bowel disease, *IBS* irritable bowel syndrome, *GI* gastrointestinal, *CRP* C- reactive protein, *ESR* erythrocyte sedimentation rate, *HIV* human immunodeficiency virus.

## Data Availability

The datasets used and/or analyzed during the current study are available from the corresponding author on reasonable request.

## Abbreviations

CRE: Carbapenem-resistant Enterobacteriaceae
HSCT: Hematopoietic stem cell transplantation
allo-HSCT: Allogeneic hematopoietic stem cell transplantation
FMT: Fecal microbiota transplantation;
CDI: Clostridium difficile infection
GVHD: Graft-versus-host disease
MDRB: Multidrug-resistant bacteria
AML: Acute myeloid leukemia
MRD: Minimal residual disease
CRKp: Carbapenem-resistant *Klebsiella pneumonia*
CT: Computerized tomography
GMP: Good manufacturing practice
AEs: Adverse events
SCFAs: Short-chain fatty acids
VRE: Vancomycin-resistant enterococci
SAEs: Serious adverse events
M: Male
F: Female
aGVHD: Acute graft-versus-host disease
GI: Gastrointestinal
rCDI: Recurrent *Clostridium difficile* infection
auto-HSCT: Autogeneic hematopoietic stem cell transplantation
