# Supplementary material for: Tandem fecal microbiota transplantation cycles in an allogeneic hematopoietic stem cell transplant recipient targeting carbapenem-resistant Enterobacteriaceae colonization: a case report and literature review
Source: Eur J Med Res. 2021 Apr 28;26:37. doi: 10.1186/s40001-021-00508-8 (PMC8080403; doi:10.1186/s40001-021-00508-8)
Supplement: Supplementary file 1 — Additional file 1: Figure S1. Antibiogram of the cultured CRKp. R resistant, S sensitive. [file 40001_2021_508_MOESM1_ESM.pdf]

Klebsiella pneumoniae

Amoxicillin/Clavulanic	>=32	R	ug/ml
Ceftriaxone	>=64	R	ug/ml
Imipenem	>=16	R	ug/ml
Cefuroxime sodium	>=64	R	ug/ml
Cotrimoxazole	>=320	R	ug/ml
Cefepime	>=32	R	ug/ml
Cefoperazone/Sulbactam	>=64	R	ug/ml
Amikacin	>=64	R	ug/ml

Ertapenem	>=8	R	ug/ml
Cefoxitin	>=64	R	ug/ml
Levofloxacin	>=8	R	ug/ml
Cefuroxime	>=64	R	ug/ml
Ceftazidime	>=64	R	ug/ml
Piperacillin/Tazobactam	>=128	R	ug/ml
Tigecycline	2	S	ug/ml
